# Kissing Spine Syndrome in Adolescents: Are We Missing This?

**DOI:** 10.7759/cureus.109112

**Published:** 2026-05-18

**Authors:** Josiah J Wolf, Mutaleeb A Shobode, Adam Mansour, Asahi Murata, Toren Moore, Ramiz Memon, Heather M Richard, Kirsten Tulchin-Francis, Reid C Chambers, Allen Kadado

**Affiliations:** 1 Orthopedic Surgery, Nationwide Children’s Hospital, Columbus, USA; 2 Orthopedic Surgery, The Ohio State University College of Medicine, Columbus, USA

**Keywords:** baastrup’s disease, interspinous process distance, kissing spine syndrome, low back pain, lumbar extension pain, pediatric back pain, pelvic incidence, spinous process sclerosis

## Abstract

Aim and objective

Low back pain (LBP) is increasingly prevalent among pediatric patients but often lacks a definitive diagnosis. Kissing Spine Syndrome (KSS), or Baastrup's Disease, is well-described in adults but understudied in children. This study characterizes the clinical and radiographic features of pediatric and adolescent patients with LBP suggestive of KSS.

Methods

We conducted a retrospective comparative study, wherein we reviewed 131 patients aged 10-22 years presenting with LBP to a pediatric orthopedic clinic in 2021. Inclusion criteria were lumbar spine radiographs and clinical examination. Exclusions included prior spine surgery, inadequate imaging, spondylolysis, spondylolisthesis, degenerative disc disease, and congenital anomalies. Patients were classified into a KSS group (n=23), defined by sclerosis on adjacent spinous processes, or a non-KSS group (n=108). Data were analyzed using non-parametric statistics.

Results

The KSS group had a higher mean age (16.5±1.8 vs 15.0±2.4 years; p=0.006) and greater female predominance (84% vs 57%; p=0.020). Pain with lumbar extension occurred in 67% of KSS and 53% of non-KSS patients (p=0.217). Patients with KSS had higher pelvic incidence (53.0°±11.5 vs 45.6°±11.3; p=0.039) and reduced interspinous distances at L2-L3 (7.5±3.3 vs 10.0±4.5 mm; p=0.015) and L4-L5 (5.1±3.1 vs 6.9±3.6 mm; p=0.013). MRI demonstrated higher frequencies of spinous process edema (22% vs 5%) and interspinous ligament edema (44% vs 24%), though differences were not statistically significant.

Conclusions

KSS is an identifiable cause of pediatric LBP, particularly in adolescent female subjects and patients performing extension-based activities. Key indicators include pain with lumbar extension, adjacent spinous process sclerosis, and reduced interspinous distances. MRI trends suggest early inflammatory changes, but larger studies are needed to define its diagnostic role.

## Introduction

Low back pain (LBP) presents a challenging clinical problem and is an increasing concern in the pediatric orthopedic setting. Earlier reports demonstrated an incidence of LBP in the pediatric population between 2% and 11% [1,2]. More recent studies, however, describe a much higher incidence, around 35%, with a marked increase near age 10, likely influenced by subject recall, that continues to rise throughout adolescence [3-5]. Historically, LBP in the adolescent population has been attributed to diagnosable pathology, such as spondylolysis, spondylolisthesis, Scheuermann Kyphosis, and infectious or neoplastic etiologies [6,7]. In contrast to these findings, recent reports have not been able to identify a cause in the majority of pediatric patients with LBP [8-10].

Baastrup’s disease, or Kissing Spine Syndrome (KSS), is a recognized but underdiagnosed cause of a specific presentation of LBP, first described by Christian Ingerslev Baastrup in 1933. He observed that adjacent lumbar spinous processes (SP) may appear abnormally close together [11]. Although there is no universally accepted radiographic definition of KSS, the literature consistently describes a core set of imaging features used to identify the condition. In addition to the hallmark close approximation or contact of adjacent SP, these may include sclerosis, flattening, and enlargement of the pseudo-articulating surfaces due to chronic impingement. If obtained, magnetic resonance imaging (MRI) can reveal bony and interspinous ligament edema, as well as cystic lesions. Typical symptoms of KSS include mechanical axial LBP aggravated by extension, relieved by flexion, and point tenderness along the SP of interest [12].

As a well-described phenomenon in the adult spinal literature, KSS is thought to result from a variety of possible causes. One major thought is age-related degeneration, specifically an increase in the height and width of lumbar SP as a biological defense reaction aimed at stabilizing a collapsing anterior column [13,14]. Other theories, particularly relevant in younger patients, include mechanical overuse in lumbar extension with repetitive loading and motion [12,15]. In addition, a postural predisposition related to sagittal malalignment and altered spinopelvic parameters has been implicated as a contributor to excessive posterior element contact and subsequent degenerative change [16-18].

Although KSS is well described in the adult population, there is a paucity of literature in pediatrics, with most reports limited to isolated case studies [15,19,20]. While the condition is typically diagnosed in adults over 60 years of age [12,14,21], we hypothesize that KSS may begin developing as early as adolescence, presenting with both characteristic symptoms and early imaging findings. As such, Baastrup’s disease may represent an underrecognized cause of LBP in the pediatric population.

The purpose of this study is to evaluate the clinical and radiographic characteristics of pediatric patients with LBP, in the absence of other verified pain sources, and concomitant findings suggestive of KSS, specifically sclerosis between two or more adjacent lumbar SP and, if present, midline lumbar tenderness or pain with lumbar extension on exam. Through this analysis, we hope to elucidate early patterns of presentation and imaging findings that may facilitate recognition and management of this condition in younger populations. To our knowledge, this represents one of the largest case series evaluating KSS-suggestive findings in a pediatric cohort.

## Materials and methods

After obtaining approval from the institutional review board of the Nationwide Children's Hospital (approval no: STUDY00002280), we reviewed all the consecutive patients aged 10 to 22 years who presented with a primary complaint of LBP to our institution’s pediatric orthopedic clinics from January 1, 2021, to December 31, 2021. Patients were identified using the International Classification of Diseases, 10th Revision (ICD-10) codes for back pain. Inclusion criteria were: (1) age between 10 and 22 years, (2) lumbar back pain, (3) availability of lumbar spine radiographs for evaluation, and (4) completion of a clinical examination.

The exclusion criteria were expanded to ensure a more homogenous study population and included: (1) previous spine surgery, (2) inability to participate in the clinical examination, (3) inadequate lumbar spine radiographs, (4) diagnosis of spondylolysis or spondylolisthesis, (5) evidence of degenerative disc disease, and (6) congenital anomalies such as Bertolotti's syndrome (lumbosacral transitional vertebrae associated with LBP).

**Figure 1:**
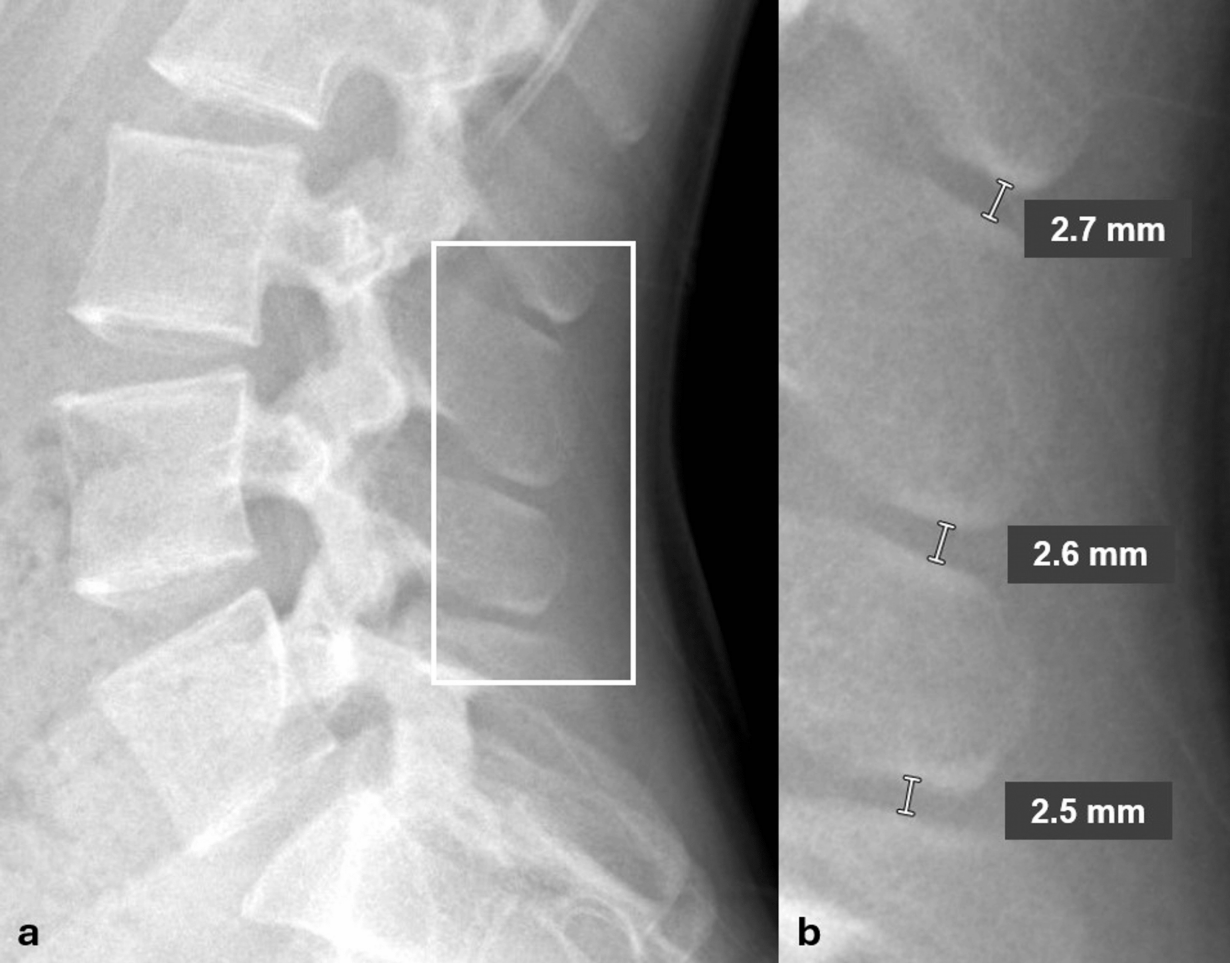
Spine radiograph depicting methodology of interspinous distance measurements (a) Lateral lumbar spine X-ray in a 15-year-old female patient demonstrating adjacent spinous process sclerosis, and (b) magnified white box denoting the methodology of measurement for interspinous distances, demonstrating reduced inter-process spaces at multiple lumbar levels.

Demographic data, including age, sex, and BMI, as well as physical examination findings and radiographic parameters, were collected from electronic medical records and radiographs. Pain levels are routinely collected using the Visual Analog Scale (VAS) at the institution, which were extracted from the chart at the time of initial LBP presentation. The KSS group was defined by the presence of sclerosis in two or more adjacent SP on standing, neutral lateral radiographs. Flexion and extension lumbar radiographs were not evaluated due to infrequent acquisition across patients. All patients underwent examination by one of six fellowship-trained pediatric orthopedic spine surgeons. Radiographic parameters, including pelvic incidence (angle between perpendicular to the sacral endplate at its midpoint and the line connecting this to the femoral head axis), lumbar lordosis (L1-S1 Cobb angle), thoracic kyphosis (T5-T12 Cobb angle), and sagittal balance (horizontal distance between C7 plumb line and posterior superior corner of S1), were measured. All interspinous distances were measured on standing lateral radiographs by one contributing author, an orthopedic research fellow, with the measurement technique confirmed by a senior author. Radiographs were viewed through the institution’s Visage Picture Archiving and Communication System (PACS) software, and the ‘Distance’ tool was used to measure the shortest distance from the inferior border of the cranial SP to the superior border of the caudal SP at each of the L2-L3, L3-L4, and L4-L5 levels (Figure 1).

Review of the MRIs was also performed by one orthopedic research fellow, specifically evaluating the lumbar spine in the sagittal plane for evidence of interspinous ligament edema and SP edema, findings not consistently characterized in radiology reports. In cases where presence of appreciable edema was uncertain, a senior author blinded to group assignment was consulted for final determination.

Statistical analysis

Descriptive statistics were performed. The Mann-Whitney U test was used to compare continuous variables, while chi-squared and Fisher exact tests were used for categorical variables. Statistical significance was set at p<0.05. Analyses were conducted using IBM SPSS Statistics for Windows, Version 29 (Released 2022; IBM Corp., Armonk, New York, United States).

## Results

Demographic data

Of the 285 patients who presented with LBP during the study period, 131 met the inclusion criteria. Among these, 23 patients (17.6%) demonstrated SP sclerosis, suggesting possible early presentation of KSS (KSS group), while the remaining 108 patients (82.4%) were assigned to the non-KSS group.

The KSS group was older (16.5 ± 1.8 years, range 13-20 vs 15.0 ± 2.4 years, range 10-22; p=0.006) and had a higher proportion of female patients compared to the non-KSS group (84% vs 57%; p=0.020). There was no significant difference in the mean body mass index (BMI) between the two groups (p=0.613) (Table 1).

**Table 1:** Demographic comparison between the kissing spine and non-kissing spine groups BMI: Body Mass Index (in kg/m^2^); Χ^2^ : Chi-square test; U: Mann–Whitney U statistic; *Statistically significant difference (p<0.05).

	Kissing spine (N=23)	Non-kissing spine (N=108)	Test statistics
Age in years, mean ± SD (range)	16.5 ± 1.8 (13-20)	15.0 ± 2.4 (10-22)	U=794.5, Z=-2.733, p=0.006*
Female, n (%)	19 (84)	61 (57)	Χ^2^ (1, N=131) =5.44, p=0.020*
BMI, mean ± SD (range)	24.0 ± 6.5 (17.2-42.7)	24.3 ± 6.1 (15.2-48.5)	U=723.5, Z=-0.506, p=0.613
BMI distribution, n (%)			
<18.5 - underweight	3/18 (17)	12/87 (14)	Χ^2^ (2, N=105) =.329, p=0.848
18.5-24.9	10/18 (56)	45/87 (52)	
>25 - overweight	5/18 (28)	30/87 (34)	

Clinical and imaging findings

Midline tenderness was present in 35% of KSS patients (n=6) and 47% of non-KSS patients (n=30; p=0.278). Pain with lumbar extension was reported more frequently in the KSS group, although the difference was not statistically significant (67% vs 53%; p=0.217). Pain scale scores and the prevalence of scoliosis were similar between groups (p=0.314 and p=0.810, respectively). In the KSS cohort, scoliosis was present in 30% of patients (vs 35% in the non-KSS cohort); most curves were mild (<25° in six of seven cases), with one patient demonstrating a 45° curve from a known prior diagnosis.

MRI utilization was higher in the KSS group (39%, 9/23) than in the non-KSS group (19%, 21/108; p=0.031). Among those who underwent MRI, SP edema was identified more frequently in the KSS group (22%, 2/9 vs 5%, 1/21; p=0.207), as was interspinous ligament edema (44%, 4/9 vs 24%, 5/21; p=0.389), although the differences were not statistically significant (Table 2). 

**Table 2:** Physical examination and comparison of imaging features between the kissing spine and non-kissing spine groups NRS: numeric rating scale, 0-10; SP: Spinous process; IP: Inter-process; MRI: Magnetic Resonance Imaging; Χ^2 : ^Chi-square test; ^1^p: Fisher exact test; U: Mann–Whitney U statistic. Continuous variables reported as mean ± standard deviations (range). Categorical variables reported as absolute numbers (%). *Statistically significant difference (p<0.05). †Patients without documentation for presence or absence of the specific exam finding were removed from the analysis.

	Kissing spine (N=23)	Non-kissing spine (N=108)	Test statistics
Midline tenderness^†^ (%)	6/17 (35)	30/64 (47)	Χ^2^ (1,N=81)=0.730, p=0.393
Paraspinal tenderness^†^ (%)	7/9 (78)	27/44 (61)	Χ^2^ (1,N=53)=0.875, p=0.349
Pain with lumbar extension^† ^(%)	10/15 (67)	28/53 (53)	Χ^2^ (1,N=68)=0.908, p=0.341
Pain level on NRS, median (IQR)	4 (2-6)	4 (1-6)	Χ^2^ (10,N=112)=11.59, p=0.314
Scoliosis (%)	7/23 (30)	38/108 (35)	Χ^2^ (1,N=131)=0.190, p=0.663
MRIs available (%)	9/23 (39)	21/108 (19)	Χ^2^ (1,N=131)=4.62, p=0.041*
SP bony edema	2/9 (22)	1/21 (5)	^1^p=0.207
Interspinous ligament edema	4/9 (44)	5/21 (24)	^1^p=0.389
Pelvic Incidence, degrees	53.0 ± 11.5 (35-71)	45.6 ± 11.3 (23-82)	U=259.0, Z=-2.06, p=0.039*
Lumbar Lordosis, degrees	58.6 ± 9.2 (44-85)	56.2 ± 16.6 (10-96)	U=1183.0, Z=-0.36, p=0.721
Thoracic Kyphosis, degrees	36.6 ± 9.9 (21-50)	35.0 ± 13.0 (5-63)	U=263.0, Z=-0.22, p=0.827
Sagittal Balance, degrees	-17.3 ± 39.3 (-73 to 37)	-23.7 ± 32.8 (-112 to 64)	U=244.0, Z=-0.40, p=0.693
L2-L3 IP distance, mm	7.5 ± 3.3 (3-15)	10.0 ± 4.5 (3-24)	U=840.0, Z=-2.43, p=0.015*
L3-L4 IP distance, mm	5.8 ± 2.4 (1-12)	7.4 ± 3.9 (1-20)	U=968.0, Z=-1.66, p=0.097
L4-L5 IP distance, mm	5.1 ± 3.1 (1-15)	6.9 ± 3.6 (2-22)	U=832.5, Z=-2.48, p=0.013*
No. of Follow-up Visits	0.3 ± 0.6 (0-2)	0.42 ± 0.75 (0-4)	U=1183.0, Z=-0.45, p=0.654

Radiographic analysis revealed a higher pelvic incidence in KSS patients (53.0° ± 11.5° vs 45.6° ± 11.3°; p=0.039), with no significant difference in lumbar lordosis, thoracic kyphosis or sagittal balance. Interspinous distance was significantly reduced in the KSS group at L2-L3 (7.5 ± 3.3 mm vs 10.0 ± 4.5 mm; p=0.015) and L4-L5 (5.1 ± 3.1 mm vs 6.9 ± 3.6 mm; p=0.013), with L3-L4 showing no significant difference (5.8 ± 2.4 mm vs 7.4 ± 3.9 mm; p=0.097).

Number of follow-up visits were infrequent for both groups (0.3 ± 0.6 visits, range 0-2 vs 0.42 ± 0.75 visits, range 0-4; p=0.654).

## Discussion

Demographics & baseline characteristics

To our knowledge, this study is the first to investigate Baastrup's Disease or KSS in a large pediatric cohort. Our findings revealed that KSS in the pediatric population may present with distinct demographic and imaging characteristics compared to non-KSS cases. The KSS group was slightly older on average than the non-KSS group; however, given the study’s focus on adolescent presentation, this difference likely reflected selection rather than a direct age effect.

More notably, the higher prevalence of female subjects in the KSS group may represent a meaningful early-stage demographic pattern of this degenerative change. This observation contrasts with existing adult KSS literature, which reports no significant sex-based differences in prevalence [21,22]. Several factors could contribute to a perceived female predilection in youth that is not sustained into adulthood. These include a generally higher prevalence of back pain among adolescent females, a greater propensity to report symptoms and seek care, or increased participation in extension-based activities (e.g., gymnastics, dance, cheerleading), as was the case for nearly half of the female patients (47%, 9/19) in our KSS cohort [1,23,24].

Our study did not find a significant correlation between BMI and KSS in adolescents, suggesting that factors other than BMI may play a more prominent role in the younger population. This contrasts with findings in adult populations, where higher BMI has been associated with an increased risk of all-cause LBP [25]. Additionally, no significant differences were observed in other baseline clinical parameters, such as VAS pain scores at presentation or the presence of concomitant scoliosis, indicating these factors are not distinguishing features between KSS and non-KSS groups. The overall scoliosis prevalence in our study population was elevated at roughly 30% compared to 2-4% in the general population, likely reflecting referral bias, as patients presenting to a tertiary orthopedic center for LBP are more likely to have underlying structural abnormalities. Furthermore, among KSS patients, spinal curvature was generally mild and appropriate for continued observation in all but one case.

Clinical findings

Although pain with lumbar extension trended toward being more frequent in the KSS group, the difference did not reach statistical significance. This may reflect the small sample size, but an equally important consideration is the limited reliability of physical examination for pediatric back pain, particularly for nonspecific findings such as midline tenderness or pain provocation [3,8]. Adult KSS studies consistently describe an anecdotal association between lumbar extension and symptom exacerbation [12,21]. Accordingly, when considered in the context of an adolescent patient’s activity history, extension-related pain remains a clinically meaningful diagnostic indicator of KSS. Thus, whether patient-reported or reproducible on exam, this finding, along with point tenderness, should continue to raise clinical suspicion.

Radiographic & MRI findings

**Figure 2:**
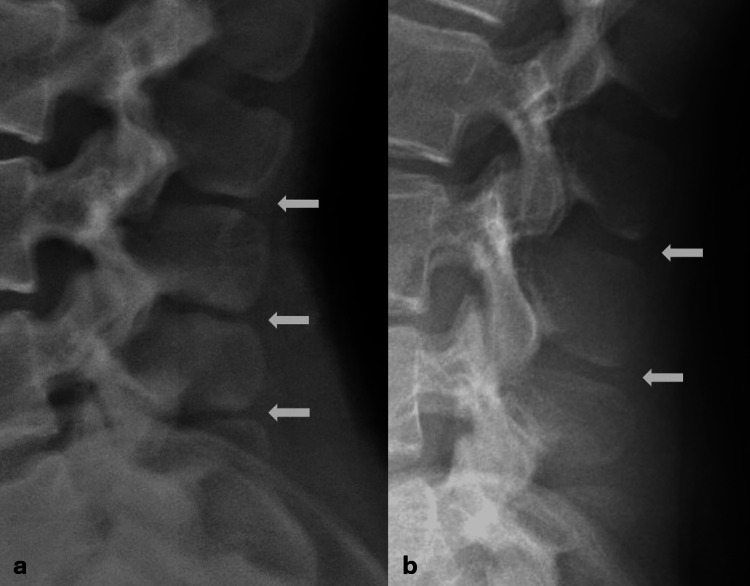
Subtle features of the Kissing Spine Syndrome in the adolescent population (a) Lateral lumbar spine X-ray in a 15-year-old female patient demonstrating spinous process sclerosis, with additional features of flattening and contour irregularity (three white arrows); (b) A second 15-year-old female patient with more subtle findings of sclerosis and flattening alone (two white arrows).

The significantly reduced interspinous distances at two of the three lumbar levels measured in our KSS group align with the radiographic hallmark of KSS; namely, the approximation or contact of adjacent SP leading to sclerosis. Importantly, this early anatomic change may act as an initial pain generator, where over time, progressive sclerosis of the articulating surfaces may worsen symptoms. Such progression may eventually contribute to interspinous bursitis and cyst formation later in the disease course [22]. In part, the challenge of identifying KSS in adolescence lies in the subtlety of SP sclerosis, but additional features of flattening, contour irregularity, or preliminary remodeling may aid recognition when present (Figure 2).

These findings highlight the importance of detailed radiographic examination when evaluating for early development of KSS.

**Figure 3:**
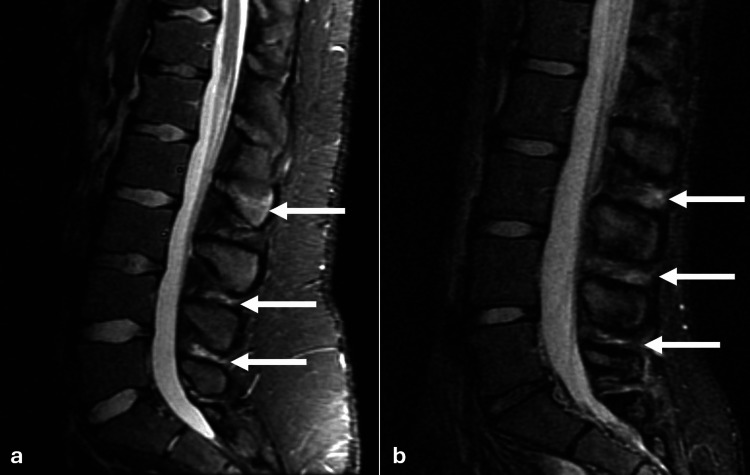
MRI findings representative of the Kissing Spine Syndrome (a) MRI, Short Tau Inversion Recovery (STIR) sequence, in a 16-year-old female patient illustrating both spinous process edema (superior white arrow) and interspinous ligament edema (two inferior white arrows); (b) An additional case of interspinous ligament edema alone (three white arrows).

Early identification of KSS may allow prevention or mitigation of more severe complications associated with long-standing disease, such as canal stenosis, disc contour abnormalities, and spondylolisthesis [22]. While KSS has characteristic radiographic features, its clinical diagnosis often requires correlation with physical examination and exclusion of other posterior element pathologies that can produce similar extension-related pain. In our cohort, MRI was obtained at the discretion of the treating clinician for a range of indications, including persistent or focal back pain, concern for posterior element stress reactions, evaluation of possible disc or ligamentous pathology, or atypical clinical presentations. As MRI was not performed uniformly in all subjects, the MRI subgroup represents a clinically selected population, where both selection and verification bias are possible. These biases may influence the observed prevalence of interspinous ligament or bony edema. In our KSS group, frequency of SP bony edema and interspinous ligament edema were 17% and 20% higher, respectively. Although statistical significance was not shown, likely secondary to limited sample size, this parallels adult studies where these MRI changes have been linked to symptomatic disease (Figure 3) [12,22].

Ultimately, we believe that MRI should not be discounted as a valuable modality when the differential diagnosis includes KSS. Further prospective studies are needed to establish its diagnostic utility in identifying early signs of KSS in the pediatric population.

We observed significant differences in pelvic incidence between the KSS and non-KSS groups. Pelvic incidence is a fundamental parameter in sagittal spinal balance and when elevated, has been linked to various spinal pathologies [26]. Although the KSS group demonstrated a higher mean pelvic incidence, the biomechanical implications of this finding remain uncertain. Any potential relationship between pelvic incidence and reduced interspinous distance is speculative, and additional biomechanical modeling or comparative studies would be needed to clarify whether a causal link exists.

Management considerations

In adults, treatment recommendations for KSS include temporary avoidance of lumbar extension activities and application of broader conservative LBP guidelines, such as nonsteroidal anti-inflammatory drugs and physical therapy focused on core strengthening and flexibility. However, the relative success of conservative treatment, both in adults and especially in pediatric patients, remains poorly defined. In adolescents, activity modification and targeted therapy may help relieve symptoms and limit progression, but our KSS cohort demonstrated limited follow-up (0.3 ± 0.6 visits, range: 0-2), preventing assessment of long-term effectiveness. Although conservative management is considered first-line, a few reported pediatric cases with persistent pain have shown improvement after interspinous ligament injections or medial branch blocks, consistent with adult experience [15,27]. Early recognition of KSS in pediatric patients is likely crucial for implementing appropriate management strategies, which could ultimately reduce the risk of more severe complications associated with long-standing disease.

Several limitations should be acknowledged. The study’s retrospective design may introduce selection bias, and the relatively small sample size of the KSS group, drawn from a single institution over one year, may limit generalizability and present referral bias. Radiographs were not magnification-corrected, as no calibration marker was present at acquisition and no automated correction was performed by the institution’s Picture Archiving and Communication System (PACS). As a result, absolute linear measurements for the interspinous distance may be influenced by geometric magnification, although all images were acquired using a consistent technique. Also, measurements were not blinded to group assignment, as the same investigator evaluating sclerosis status also measured interspinous distances. This may introduce measurement bias despite the use of standardized procedures.

Verification bias may also be present because MRI evaluation was more likely to be performed in patients with concerning clinical or radiographic findings, leading to non-random confirmation of pathology. Analysis of documented clinical exam findings was constrained by inconsistent testing for KSS-suggestive features. Reduced clinical suspicion may have stemmed from limited awareness of KSS as a differential diagnosis. Additionally, minimal follow-up in the KSS group suggests either mild, self-limited symptoms or the need for long-term monitoring to assess disease progression and treatment efficacy. Evaluating long-term outcomes of KSS development in this younger population remains an important area for future research.

## Conclusions

KSS is a diagnosable pathology in pediatric patients presenting with LBP, particularly those active in sports involving repetitive lumbar extension. Clinical features such as point tenderness in the interspinous space and pain with extension activities, along with radiographic findings of adjacent SP sclerosis and reduced interspinous distances, are indicative of KSS in this population. Female gender may be an important early demographic association, while relative age, interpreted alongside imaging findings, should not preclude consideration of KSS in the evaluation of pediatric LBP. Although MRI comparisons did not reach statistical significance, observed trends of increased SP and interspinous ligament edema highlight the need for larger studies to clarify the role of advanced imaging in early detection. Timely recognition and conservative management may help mitigate progression and improve outcomes. Future research should examine the biomechanical, athletic, and developmental factors contributing to KSS in adolescents to enhance diagnostic accuracy and guide treatment strategies.
